# Supplementary material for: Maternally Inherited Differences within Mitochondrial Complex I Control Murine Healthspan
Source: Genes (Basel). 2019 Jul 13;10(7):532. doi: 10.3390/genes10070532 (PMC6678443; doi:10.3390/genes10070532)
Supplement: Supplementary file 1 [file genes-10-00532-s001.zip › Supplementary file/Supplementary_materials_proof.docx]

**Supplemental materials**

Supplemental materials and methods

References for supplemental experimental procedures

Figure S1: Data related to Figure 1.

Figure S2: Data related to Figure 3.

Figure S3: Data related to Figure 4.

Figure S4: Data related to Figure 5.

Table S1: List of mtDNA variations in *C57BL/6J-mt^BPL/1J^* and *C57BL/6J-mt^ALR/LtJ^*.

Table S2: Nuclear genome homology of B6-mt^ALR^ and B6-mt^BPL^ mice to B6.

Table S3: The statistical analysis for the lifespan of B6-mt^BPL^ and B6-mt^ALR^ mice.

Data file S1 (xlsx.): DEG (Differentially expressed genes) B6-mt^ALR^ vs B6-mt^BPL^.

**Supplemental materials and methods**

**Cell culture**

**Fibroblast cell line generation.** Primary skin fibroblasts were prepared from female B6-mt^ALR^ and B6-mt^BPL^ mice as previously described^1^, and the cell samples (passage 1) were sent to InSCREENeX (InSCREENeX GmbH, Braunschweig, Germany) and immortalised to generate conplastic fibroblasts carrying respective mutations in mtDNA. The mtDNA of the generated cell lines were deep-sequenced using Illumina MiSeq platform as previously described^2^, and the variations were identical to each conplastic mouse strain as shown in **Table S1**.

**Genotyping**

**Next generation sequencing of whole mitochondrial genome.** Total DNA was isolated from conplastic skin fibroblast cell lines, and deep-sequencing for the whole mtDNA was performed as previously described^2^.

**Nuclear genome SNP genotyping.** Total tail DNA samples were genotyped for 77,808 SNP markers in nuclear genome using the Mouse Universal Genotyping Array (MegaMUGA, GeneSeek Europe, Scotland, UK). Array processing and genotype calling were performed by GeneSeek as previously described^3^.

**Mitochondrial functional studies**

**Measurement of hydrogen peroxide.** Hydrogen peroxide content in liver mitochondrial was measured using Amplex Red H_2_O_2_ assay kit (Thermo Fisher Scientific).

**Mitochondrial superoxide and mitochondrial membrane potential measured in primary mouse lymphocytes.** Mitochondrial superoxide and mitochondrial membrane potential (MMP) were assessed in primary lymphocytes immediately after the preparation and after 24 hours activation with anti-CD3 and anti-CD28 antibodies (eBioScience). Cells were washed with 2 % FBS in PBS (FACS buffer) and stained with 5µM MitoSOX Red (Invitrogen) for 10 minutes at 37 °C, followed by Annexin V-APC (eBioScience) staining for 10 min at room temperature and measurement. For MMP, cells were stained with 300 nM of tetramethylrhodamine, ethyl ester (TMRE, Invitrogen) and 200nM of MitoTrackerGreen (Invitrogen) for 15 min at 37 °C and washed with FACS buffer twice. Signal was detected using FACSCalibur (BD Bioscience). Data was analysed using the software FlowJo, and the geometric means of the MitoSOX signal in the Annexin V-negative lymphocyte population were taken as the MitoSOX value, and the geometric mean of TMRE signal in the MitoTrackerGreen-positive viable cell population was taken as the MMP value.

**Mitochondrial respiratory chain complex activities.** Mitochondrial respiratory chain complex activities were measured in liver-isolated mitochondria obtained from both B6-mt^ALR^ and B6-mt^BPL^ female mice as previously described^2^.

**Mitochondrial oxygen consumption (Seahorse XF analyzer).** To evaluate the metabolic phenotype of skin fibroblast cell lines and primary hepatocytes isolated from mice, oxygen consumption (OCR, in pmol/min) was measured using the Seahorse XF24 analyzer (Agilent, North Billerica, USA) as previously described^4^. Primary mouse hepatocytes were prepared as previously described, and hepatocytes were plated at 10^4^ cells per well, and cultured in high glucose DMEM (4.5 g/L) in a gelatine-coated 24-well Seahorse XF24 cell culture plate overnight, and the assay was performed on the following day of the preparation. Conplastic fibroblast cell lines were resuspended into DMEM, plated onto a 24-well Seahorse XF24 cell culture plate at 10^4^ cells per well, and cultured overnight to allow cells to attach the plate. Measurement was started after replacing the medium into XF MitoStress assay buffer (DMEM without bicarbonate supplemented with 4.5 g/L D-glucose, 2 mM L-glutamine, 1mM pyruvate, pH 7.4). After base line measurement, 1µM oligomycin was added to inhibit the ATP synthase, thereby determine ATP-linked oxygen consumption and proton leak. Next, 0.4 µM FCCP was administrated to uncouple the mitochondrial membrane potential, allowing maximum electron flux to determine maximal respiration and spare capacity. Finally, a mixture of 1 µM antimycin A and 1 µM rotenone was added to completely block mitochondrial electron transport and define the non-mitochondrial oxygen consumption. Calculation of basal oxygen consumption, OXPHOS-linked ATP production, maximal respiration, spare capacity, and non-mitochondrial respiration values were conducted according to the previous report^5^.

**NAD^+^ and NADH determination in liver tissues.** NAD^+^ and NADH were measured in mouse liver tissues using Amplite^TM^ NAD^+^/NADH Ratio Assay Kit (AAT Bioquest®, Inc. Sunnyvale, CA, USA), according to the manufacturer’s protocol.

**Analysis of tryptophan levels in fibroblasts using nuclear magnetic resonance (NMR).** Conplastic skin fibroblast cell lines carrying either *mt-Nd2* or *mt-Nd5* mutations were prepared to be used for NMR analysis. Sample preparation was performed as previously described^6^. All steps have been performed at 4°C. In brief, the cell pellets were lysed by adding 900 µl of a 2:1 mixture of chloroform/methanol and sonication. An equal volume of chloroform saturated water was added and after phase separation by centrifugation the upper aqueous phase was lyophilized and stored at -80 °C. The water-soluble metabolites were dissolved in 180 µl of 100 mM sodium phosphate buffer, 0.1 mM TSP-*d*_4_, pH 7.4 in D_2_O. NMR measurements (pulse sequence noesypr1d) were performed on a Bruker Avance 500 equipped with a TCI cryo probe at 298 K system temperature (Bruker, Massachusetts, USA). The residual water signal was suppressed by saturation during the relaxation delay (3 s) and the mixing time (0.1 s). The spectra have been manually phased, base line corrected and referenced via the TSP-*d*_4_ main signal at 0 ppm. Regions containing resonances of water (4.54 – 5.2 ppm), chloroform (7.66 – 7.7 ppm), DMSO (2.725 – 2.75 ppm), methanol (3.329 – 3.382 ppm), ethanol (1.16 – 1. 214 ppm, 3.575 – 3.713 ppm), and TSP-*d*_4_ (-0.2 – 0.2 ppm) were excluded before normalization of the spectra and subsequent data analysis of the tryptophan levels.

**Western blotting.** Liver and heart tissues were prepared as previously described^2^. Western blotting was performed with antibodies against OXPHOS proteins (MitoProfile Total OXPHOS Rodent WB Antibody Cocktail; Abcam, Cambridge, UK), NDUFA1 (rabbit polyclonal, Abcam), NDUFS3 (17D95, Abcam), GRIM19 (6E1BH7, Abcam), and VDAC (rabbit polyclonal, EMD Millipore, Darmstadt, Germany) for heart protein, and with those against HSP60 (N-20, Santa Cruz Biotechnology, Inc., Heidelberg, Germany) and HSP90 (Clone 68, BD Biosciences, Heidelberg, Germany) for liver protein. HRP-labelled anti-goat IgG, anti-rabbit IgG and anti-mouse IgG (Agilent Technologies, Hamburg, Germany) were used as secondary antibodies.

**Metabolic phenotyping**

**High fat diet feeding experiment.** Feeding scheme was described in Mice and husbandry. Body mass of each mouse was evaluated weekly, and the intraperitoneal glucose tolerance test was conducted at 8 weeks of feeding.

**Intraperitoneal glucose tolerance test (IPGTT).** Mice were fasted for 18 hours and then glucose (2 g/kg) was intraperitoneally injected. Glucose was determined from tail vein blood before and 15, 30, 45, 60 and 120 min after glucose administration using a glucometer (ACCU-CHEKR Performa, Roche Diagnostics GmbH, Mannheim, Germany).

**Plasma parameter assay.** Determination of glucose, fructosamine, free fatty acids and total cholesterol, HDL and LDL in plasma was performed using an automated analyzer (Cobas Mira S, Hoffmann-La Rosche, Basel, Switzerland) with the appropriate commercially available reagent kits (glucose HK, fructosamine, cholesterol PAP, Diatools, Villmergen, Switzerland; and NEFA HR, Wako, Neuss, Germany). Plasma insulin levels were measured using an immunoassay-based Mouse/Rat Insulin Kit on a Sector Imager (Meso Scale Discovery, Gaithersburg, MD, USA) according to the manufacturer’s instructions.

**Indirect calorimetric cage analysis.** Oxygen consumption, carbon dioxide production, food and water intake, and locomotor activity were continuously monitored using an open-circuit indirect calorimetry system (PhenoMaster System^TM^, TSE, Bad Homburg, Germany) as previously described^2^.

**Whole transcriptome analysis.** Total RNA was prepared from liver tissue using innuPREP RNA Mini Kit (Analytik Jena AG, Jena, Germany). Library preparation and deep sequencing was performed by Chronix Biomedical (Göttingen, Germany) on the Illumina platform. Fastq reads were pseudo-aligned to the mm10 genome assembly using Kallisto^7^ and transcript read counts were aggregated to Ensembl Gene IDs for further analysis. Differential gene expression analysis was performed via the R library sleuth^8^ using a linear model that accounted for mouse age and strain. Significance and effect sizes of differential gene regulation were calculated from the likelihood ratio and the Wald test, respectively, as implemented in the sleuth package. GO term and pathway enrichment analyses were performed based on the effect size between the different mouse strains or age using the generally applicable Gen Set Enrichment Analysis (GSEA), GAGE, which determines whether a set of genes is systematically up- or down-regulated as a whole ^9^. For gene set definitions, we used the Molecular Signatures Database (MSigDB) collections v.5.2 that were mapped to mouse ortholog Entrez IDs via the HGNC Comparison of Orthology Predictions (HCOP) (provided by the Walter and Eliza Hall Institute; <http://bioinf.wehi.edu.au/software/MSigDB/>). Gene sets with less than 10 or those with more than 500 members were discarded for statistical robustness and biological interpretation.

**Data availability.** RNA-seq data has been uploaded to Gene Expression Omnibus (GEO) with the ID GSE114256.

**References for supplemental experimental procedure**

1. Schauer, M. *et al.* A mutation in the NADH-dehydrogenase subunit 2 suppresses fibroblast aging. *Oncotarget* **6**, 8552–8566 (2015).

2. Hirose, M. *et al.* Low-level mitochondrial heteroplasmy modulates DNA replication, glucose metabolism and lifespan in mice. *Sci Rep* **8**, 5872 (2018).

3. Collaborative Cross Consortium. The genome architecture of the Collaborative Cross mouse genetic reference population. *Genetics* **190**, 389–401 (2012).

4. Hirose, M. *et al.* Mitochondrial gene polymorphism is associated with gut microbial communities in mice. *Sci Rep* **7**, 15293 (2017).

5. Dott, W., Mistry, P., Wright, J., Cain, K. & Herbert, K. E. Modulation of mitochondrial bioenergetics in a skeletal muscle cell line model of mitochondrial toxicity. *Redox Biol* **2**, 224–233 (2014).

6. Richer, B. C., Salei, N., Laskay, T. & Seeger, K. Changes in Neutrophil Metabolism upon Activation and Aging. *Inflammation* **41**, 710–721 (2018).

7. Bray, N. L., Pimentel, H., Melsted, P. & Pachter, L. Near-optimal probabilistic RNA-seq quantification. *Nat. Biotechnol.* **34**, 525–527 (2016).

8. Pimentel, H., Bray, N. L., Puente, S., Melsted, P. & Pachter, L. Differential analysis of RNA-Seq incorporating quantification uncertainty. *bioRxiv* (2016). doi:https://doi.org/10.1101/058164

9. Luo, W., Friedman, M. S., Shedden, K., Hankenson, K. D. & Woolf, P. J. GAGE: generally applicable gene set enrichment for pathway analysis. *BMC Bioinformatics* **10**, 161 (2009).

**Supplemental figures**

**Figure S1**

**A B**

**** **
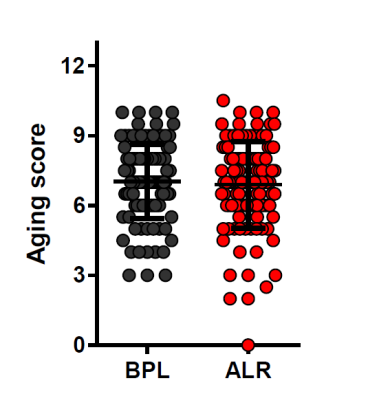
**

**C D**

**Figure S1: Date related to Figure 1.**

**A.** Spontaneous disease incidence in the lifespan study. The incidence of ulcerative dermatitis (UD), tumour, and arthritis were comparable between the strains. Sex-mixed analysis. One hundred seventy-six B6-mt^BPL^ and 181 B6-mt^ALR^ mice were evaluated for the ulcerative dermatitis and arthritis and 127 B6-mt^BPL^ and 137 B6-mt^ALR^ mice for tumour. BPL: B6-mt^BPL^; ALR: B6-mt^ALR^.

**B.** Ageing score evaluated in 112 B6-mt^BPL^ and 118 B6-mt^ALR^ mice at the moribund stage did not show the difference. Sex-mixed analysis.

**C.** Sexual maturity observed in 44 B6-mt^BPL^ and 38 B6-mt^ALR^ mice was comparable between strains, with a trend of earlier sexual maturity in B6-mt^ALR^ mice. VP; vaginal patency.

**D.** Plasma levels of IGF-1 exhibited age-related increased levels, with a trend of higher levels in aged B6-mt^ALR^ than B6-mt^BPL^.

**Figure S2**

**Figure S2: Data related to Figure 3**

NAD^+^, NADH, and total NADH and NAD^+^ values measured in the same samples tested in **Figure 3C**. The ratio of NAD^+^ to NADH presented in **Figure 3C** was calculated with the values in this figure. BPL: B6-mt^BPL^; ALR: B6-mt^ALR^.

**Figure S3**

**A**

**B**

**Figure S3: Data related to Figure 4.**

Gene set enrichment analysis of genes whose expression was affected by the *mt-Nd2* and *mt-Nd5* variation. The network represents the significant gene sets as nodes; with the node colour corresponding to the significance and the node diameter to the gene background indicate the most prominent function in the clustered nodes. Up-regulated pathways in the shorter-lived B6-mt^ALR^ (**A**), and up-regulated pathways in the longer-lived B6-mt^BPL^ mice (**B**).

**Figure S4**

**A B**

**C**

**** ****

**D**

**** ****

**E F**

**Figure S4 (cont.)**

**G H**

**
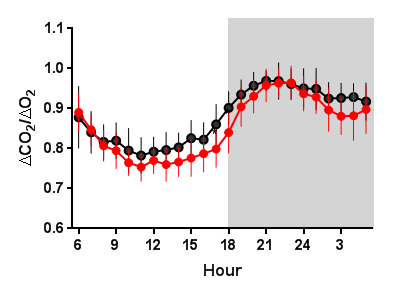
**
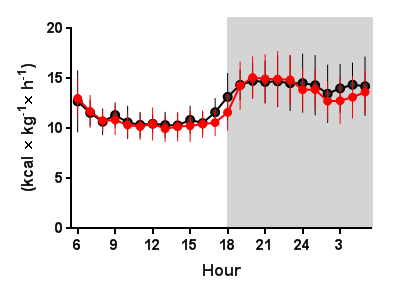


**I J**

**
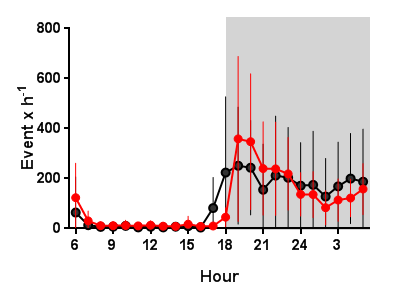
**

**K**

**
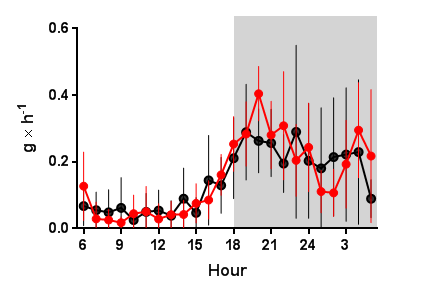
**

**Figure S4: Data related to Figure 5.**

**A.** Glucose and insulin levels were determined in morning-fasted control diet (CD)-fed female B6-mt^BPL^ and B6-mt^ALR^ mice. BPL: B6-mt^BPL^; ALR: B6-mt^ALR^.

**B.** Glucose, insulin and fructosamine levels were determined in random-fed female B6-mt^BPL^ and B6-mt^ALR^ mice.

**C, D.** Lipid parameters were measured in CD-fed female B6-mt^BPL^ and B6-mt^ALR^ (**C**), and random-fed female B6-mt^BPL^ and B6-mt^ALR^ (**D**).

**E.** The IPGTT result of control diet (CD; 13 kJ% fat)-fed mice. B6-mt^ALR^ mice showed mild, but impairment of glucose tolerance compared to B6-mt^BPL^ mice. Main effect of strains: descriptive *p* = 0.0062, interaction between groups and time: descriptive *p* = 0.0017, two-way non-parametric analysis of variance.

**F.** IPGTT was performed independently from the diet-experiment. Regular chow-fed mice were tested twice, and the presented graph includes data from the 2 independent experiments using 3 to 4 months old female mice. Main effect of strains: descriptive *p* = 0.3062, interaction between strains and time: descriptive *p* = 0.0056, two-way non-parametric analysis of variance.

**G-K.** Indirect calorimetric cage analysis of young (3–5 months old) female mice. Respiratory exchange ratio (G), energy expenditure (H), locomotor activity (I), food intake (J) and water intake (K) were evaluated, and under the curve (AUC) was compared between the strains. The left panels: descriptive *p* = 0.0503 (G); descriptive *p* = 0.0094 (J), two-way ANOVA. The right panels (AUC): descriptive *p* = 0.0382 (G); descriptive *p* = 0.0167 (J); *t-*test. *n* = 10 (B6-mt^BPL^); *n* = 12 (B6-mt^ALR^).

**Supplemental tables**

**Table S1: List of mtDNA variations in *C57BL/6J-mt^BPL/1J^* and *C57BL/6J-mt^ALR/LtJ^*.**

| Position | 4738 | 11902 |
| --- | --- | --- |
| Strain \| Gene | *mt-Nd2* | *mt-Nd5* |
| *C57BL/6J-mt^BPL/1J^* | C | C |
| *C57BL/6J-mt^ALR/LtJ^* | A | T |
| AA change | Leu-Met | Phe-Leu |

**Table S2: Nuclear genome homology of B6-mt^ALR^ and B6-mt^BPL^ mice to B6.**

| Strain | | | B6-mt^ALR^ | | | B6-mt^BPL^ | | |
| --- | --- | --- | --- | --- | --- | --- | --- | --- |
| ID | | | 39236 | 39178 | 54917 | 35811 | 31911 | 39444 |
| Sex | | | M | F | F | M | F | F |
| ChrB37 | PosB37 | SNPs |  |  |  |  |  |  |
| 1 | 156715218 | UNC010465120 | TT | TT | TT | ***TG*** | ***TG*** | TT |
| 3 | 84097284 | UNC030314030 | GG | GG | GG | ***N.D.**** | GG | GG |
| 3 | 120369799 | B6_03-120369799-S | AA | AA | AA | AA | ***N.D.**** | AA |
| 3 | 121531310 | UNC030194728 | CC | CC | CC | CC | ***CT*** | CC |
| 5 | 118166937 | B6_rs29730106 | AA | AA | AA | ***AG*** | ***AG*** | AA |
| 5 | 136917666 | B6_rs29500641 | TT | TT | TT | ***TG*** | ***N.D.**** | TT |
| 9 | 6238770 | B6_09-006238770-S | AA | AA | AA | ***AG*** | ***AG*** | AA |
| 11 | 87932613 | UNC20071212 | CC | CC | CC | ***N.D.**** | AA | AA |
| 11 | 120428220 | UNC20527000 | TT | ***N.D.**** | TT | ***TC*** | ***N.D.**** | ***N.D.**** |
| 14 | 22151051 | B6_rs31151615 | TT | TT | TT | ***TC*** | ***N.D.**** | TT |
| 14 | 22662231 | UNC140101805 | AA | AA | AA | ***AC*** | ***AC*** | AA |
| **nDNA homology to *C57BL/6J* (%)** | | | **100** | **99.999** | **100** | **99.988** | **99.988** | **99.999** |

*N.D.; no data due to the genotyping errors.

**Table S3: The statistical analysis for the lifespan of B6-mt^BPL^ and B6-mt^ALR^ mice.**

1. **Statistical analysis for survival curves; B6-mt^BPL^ versus B6-mt^ALR^.**

|  | *p*-value (log-rank) | *p*-value (Gehan) |
| --- | --- | --- |
| Total | 0.153 | 0.044 |
| Female | 0.040 | 0.00294 |
| Male | 0.492 | 0.874 |

1. **Survival times by sex.**

|  | N | Events | Median survival (days) | 95% CI |
| --- | --- | --- | --- | --- |
| Female | 164 | 164 | 800 | 774-832 |
| Male | 193 | 193 | 855 | 843-874 |

1. **Median survival times by strain.**

|  | N | Events | Median survival (days) | 95% CI |
| --- | --- | --- | --- | --- |
| B6-mt^BPL^ | 176 | 176 | 844 | 826-870 |
| B6-mt^ALR^ | 181 | 181 | 832 | 795-852 |

1. **Median survival times by strain and sex.**

|  | Sex | N | Events | Median survival (days) | 95% CI |
| --- | --- | --- | --- | --- | --- |
| B6-mt^BPL^ | Male | 94 | 94 | 852 | 829-896 |
|  | Female | 82 | 82 | 829 | 801-870 |
| B6-mt^ALR^ | Male | 99 | 99 | 856 | 843-885 |
|  | Female | 82 | 82 | 772 | 741-806 |
